# Antifeedant, larvicidal and growth inhibitory bioactivities of novel polyketide metabolite isolated from *Streptomyces* sp. AP-123 against *Helicoverpa armigera* and *Spodoptera litura*

**DOI:** 10.1186/1471-2180-13-105

**Published:** 2013-05-13

**Authors:** Mariadhas Valan Arasu, Naif Abdullah Al-Dhabi, Valsalam Saritha, Veeramuthu Duraipandiyan, Chinnasamy Muthukumar, Sun-Ju Kim

**Affiliations:** 1 Department of Bio-Environmental Chemistry, Chungnam National University, 99 Daehak-Ro, Yuseong-Gu, Daejeon 305-764, Republic of Korea; 2 Global Research Centre for Biotechnology, Taramani, Chennai, India; 3 Department of Botany and Microbiology, Addiriyah Chair for Environmental Studies, College of Science, King Saud University, P. O. Box 2455, Riyadh, 11451, Saudi Arabia

**Keywords:** *Streptomyces* sp. AP-123, Polyketide metabolite, Antifeedant, Larvicidal, *Helicoverpa armigera*, *Spodoptera litura*

## Abstract

**Background:**

Considerable attention has been paid to actinomycetes, especially the secondary metabolites obtained from *Streptomyces* species, as the best alternatives to chemicals as biological control agents for polyphagous pests such as *Helicoverpa armigera* and *Spodoptera litura.* On the basis of their novel biocontrol attributes, novel polyketide metabolite isolated from marine *Streptomyces* sp. AP-123 exhibited significant antifeedant, larvicidal and growth inhibitory activities against polyphagous pests.

**Results:**

Leaf disc no-choice method was used for the insect bioassay. The polyketide metabolite presented significant antifeedant activities against *H. armigera* (78.51%) and *S. litura* (70.75%) at 1000 ppm concentration. The metabolite also exhibited high larvicidal activities against *H. armigera* (63.11%) and *S. litura* (58.22%) and the LC_50_ values were 645.25 ppm for *H. armigera* and 806.54 ppm for *S. litura*. The metabolite also prolonged the larval–pupal duration of the insects at all the tested concentrations.

**Conclusions:**

The activities of the polyketide metabolite were concentration dependent for both the insects therefore it could be used as an agent to prepare new pesticidal formulations.

## Background

According to the report of FAO, US $120 billion losses worldwide were caused by 20–40% decrease in crop yield, due to the attack from pathogenic organisms and insect pests [1]. *Helicoverpa armigera* and *Spodoptera litura* are the major polyphagous pests attacking more than 150 different host species and affect the vegetable yield [2]. Therefore these pests are considered as the most economically important insect pests in many countries including India, Japan, China and Southeast Asia. Controlling these polyphagous pests becomes the challenging work in agriculture field. There are few chemical insecticides and pesticides are commercially available in the market. Usage of different varieties of chemical insecticides and pesticides to control insects has resulted in emergence of pesticide resistance in the pests [3]. To ensure the stable and high output of crops, huge amount of pesticides were applied to control the pests, and this not only caused serious environmental pollution but also induced in a wide range of pesticide resistance. Meanwhile by applying these chemical pesticides different varieties of pest predators were killed and the ecological balance was destroyed, thereby causing pest resurgence and a greater outbreak of secondary pests [4]. Due to this reason, many researchers have involved on alternative control methods. Botanical and microbial pesticides are having advantage over chemical pesticides by its highly effective, safe, and ecologically acceptable nature. Fortunately, bio-pesticides have been gaining increased attention and interest among those concerned with developing environment friendly and safe integrated crop management, with compatible approaches and tactics for pest management [5].

Natural products derived from plants and microorganisms have been used for insect control [6]. Azadirachtin, a natural compound isolated from neem *Azadirachta indica*, is considered superior over other compounds since it has wide range of biological activities. Azadirachtin has been studied by many researchers and used as positive control. Bacterial and viral-based insecticides controlled different pests. Most of the pesticides from microorganisms have been isolated from entomo-pathogens and the terrestrial environment [7]. Recent studies on marine microorganisms have focused mainly on the discovery of human drugs, whereas limited information about marine microorganisms possessing insecticidal activities has been reported. However marine environment, representing more than two thirds of our planet, is still under-explored and is considered to be a prolific resource for the isolation of less exploited microorganisms [8]. The ocean is a resource of huge drug, where more than 6000 kinds of novel chemical compounds have been isolated from marine living organisms, among which more than 1000 compounds exert biological activities, such as anti-tumour, anti-microbial and anti-virus, etc. [9].

**Figure 1:**
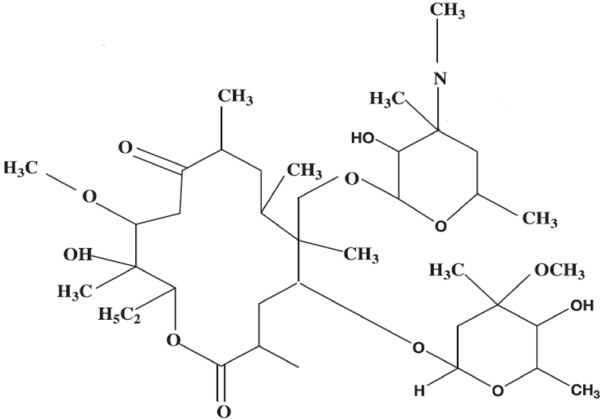
**Polyketide antimicrobial metabolite isolated from *Streptomyces *sp. AP-123 **[10]**.**

Recently, *Streptomyces* sp. AP-123 producing polyketide metabolite (Figure 1) was reported by analyzing the presence of polyketide biosynthesis (PKS) biosynthetic cluster [10]. *Streptomyces* sp. AP-123, a Gram positive, filamentous, spore-forming antagonistic bacteria recovered from marine region at Andhra Pradesh, India. Polyketide metabolite isolated from *Streptomyces* sp. AP-123 acted as a growth inhibitor of Gram-positive, Gram-negative bacteria and filamentous fungi. No reports are available on the effect of polyketide metabolite against the polyphagous pest *H. armigera* and *S. litura*. The present study was aimed at assessing the antifeedant, larvicidal, pupicidal and growth inhibitory effect of polyketide metabolite isolated from *Streptomyces* sp. AP-123 against *H. armigera* and *S. litura* .

## Results and discussion

In the present study, polyketide metabolite derived from *Streptomyces* sp. AP-123 revealed strong antifeedant activity of 78.51% and 70.75% against *H. armigera* and *S. litura*, respectively at 1000 ppm concentration and the activity was statistically significant over control (P ≤ 0.05) (Table 1). The bioactivity was directly proportional to the concentration of the metabolite. Polyketide metabolite showed 68.41% and 60.02% larvicidal activities against *H. armigera* and *S. litura*, respectively at 1000 ppm and the activity was statistically significant compared to control (P ≤ 0.05) (Table 2). The metabolite exhibit marked toxicity effect on the larvae of *H. armigera* and *S. litura*. The larvae which had consumed less amount of treated diet showed higher amount of larval mortality. The LC_50_ and LC_90_ values were 645.25 and 1724.58 ppm and 806.54 and 1725.50 ppm for *H. armigera* and *S. litura*, respectively.

**Table 1:** **Antifeedant activity of polyketide metabolite against *H. armigera *and *S. litura***

**Concentration (ppm)**	**Antifeedant activity (%)**	
	***H. armigera***	***S. litura***
*Polyketide metabolite*		
125	38.01 ± 2.11^b^	35.93 ± 3.14^b^
250	51.77 ± 3.81^c^	46.19 ± 3.88^c^
500	64.29 ± 3.78^d^	59.58 ± 2.41^d^
1000	78.51 ± 3.90^e,f^	70.75 ± 2.46^e,f^
*Azadirachtin*		
125	62.20 ± 3.05^d^	65.47 ± 2.92^e^
250	64.37 ± 3.26^d,e^	75.41 ± 5.34^f^
500	74.51 ± 4.95^f^	83.73 ± 3.53^g^
1000	89.84 ± 5.65^g^	89.61 ± 2.88^h^
Control	4.33 ± 1.07^a^	1.54 ± 1.04^a^

Mean ± SD within columns followed by the same letter do not differ significantly using Tukey’s test, P ≤0.05.

**Table 2:** **Larvicidal(%) and effective concentrations (LC**_**50 **_**and LC**_**90 **_**ppm) of polyketide metabolite against *H. armigera *and *S. litura***

**Concentration (ppm)**	***H. armigera***			***S. litura***		
	**Larvicidal (%)**	**LC**_**50**_	**LC**_**90**_	**Larvicidal (%)**	**LC**_**50**_	**LC**_**90**_
*Polyketide metabolite*						
125	15.52 ± 5.29^a^			10.44 ± 0.60^a^		
250	33.16 ± 4.34^b^	645.25	1724.58	29.11 ± 4.11^b^	806.54	1725.01
500	54.08 ± 5.63^c^			47.77 ± 3.04^c^		
1000	68.41 ± 6.04^d^			60.02 ± 2.43^d^		
*Azadirachtin*						
125	47.77 ± 4.26^c^			51.98 ± 5.95^c,d^		
250	63.66 ± 4.47^d^	170.48	401.65	69.18 ± 6.42^e^	135.58	452.02
500	98.77 ± 4.45^e^			95.77 ± 5.18^f^		
1000	100 ± 00^e^			100 ± 00^f^		

Mean ± SD within columns followed by the same letter do not differ significantly using Tukey’s test, P ≤ 0.05.

Table 3 shows pupicidal activity of polyketide metabolite consumed larvae of *H. armigera* and *S. litura*, respectively. After treatment with polyketide metabolite the larval and pupal developmental periods were increased significantly. The interference of toxic substances in the moulting process triggers the larval duration. Due to the treatment of the compound; larvae become small in size and various kinds of abnormalities were observed, therefore the larvae were not able to go into further instars. The larvae were unable to continue normal physiological processes since the larvae consumed very low amount of diet. Moulting was also delayed. Larval developmental period was increased in treatment (13.98 and 13.96 d) when compared to control (9.08 and 8.95 d) for *H. armigera* and *S. litura*, respectively. Pupal duration was also increased in treatment (15.45 and 14.4 d) when compared to control (9.58 and 11.12 d) for *H. armigera* and *S. litura*, respectively. The metabolite showed pupicidal activities of 62.01% and 55.06% against *H. armigera* and *S. litura*, respectively at 1000 ppm concentration (Table 3). Pupicidal activities were statistically significant with increasing concentrations of the compound. In general, prolonged larval–pupal durations were directly proportional to the increase in pupicidal activities. Treatment produced different kinds of abnormalities such as larval–pupal, pupal–adult intermediate and adult abnormalities were also observed.

**Table 3:** **Growth inhibitory activity of polyketide metabolite against *H. armigera *and *S. litura***

**Concentration (ppm)**	***H. armigera***					***S. litura***				
	N*	**Larval duration (d)**	**Pupicidal (%)**	N*	**Pupal duration (d)**	N*	**Larval duration (d)**	**Pupicidal (%)**	N*	**Pupal duration (d)**
*Polyketide metabolite*										
125	42	10.09 ± 0.44^b^	20.99 ± 4.15^b^	33	11.45 ± 0.40^b^	43	10.02 ± 0.29^a,b^	18.51 ± 6.33^b^	35	10.28 ± 0.22a
250	33	10.91 ± 0.35^b,c^	32.58 ± 5.20^b,c^	24	12.35 ± 0.46^b,c^	34	10.44 ± 0.87^b^	25.06 ± 7.22^b^	25	11.53 ± 0.69b
500	24	12.55 ± 0.37^c^	42.55 ± 3.47^c^	14	13.50 ± 0.70^c^	21	11.96 ± 0.45^c^	47.13 ± 10.9^c^	11	13.86 ± 0.63c
1000	18	13.98 ± 0.51^d^	62.01 ± 11.7^d^	8	15.45 ± 1.03^d^	18	13.96 ± 0.92^c^	55.06 ± 9.12^c^	8	14.4 ± 0.54cd
*Azadirachtin*										
125	26	14.09 ± 0.16^e^	70.45 ± 9.04^d^	8	17.95 ± 0.54^e^	23	14.56 ± 0.26^d,e^	47.40 ± 7.48^c^	12	14.10 ± 0.48c
250	17	15.8 ± 0.74^f^	100 ± 00^e^			15	15.95 ± 0.98^e^	76.08 ± 12.9^d^	4	15.24 ± 0.5d
500	0									
1000										
Control	48	9.08 ± 0.15^a^	0^a^	48	9.58^a^	48		8.95 ± 0.49^a^	48	11.12 ± 0.39a

Mean ± SD within columns followed by the same letter do not differ significantly using Tukey’s test, P ≤ 0.05. N*: number.

In the present study, polyketide metabolite exhibited maximum antifeedant activity of 78.51% and 70.75% at 1000 ppm concentration against *H. armigera* and *S. litura*. This result coincided with earlier results of Kannan who had isolated violacein from *Chromobacterium violaceum* claimed more than 80% antifeedancy at 1000 ppm against *H.armigera *[11]. Xiang et al. isolated novel macrocyclic lactone from *Streptomyces microflavus* neau3, showed high acaricidal activity against adult mites and nematocidal activity against *Caenorhabditis elegans *[12].

In the present study, significant larvicidal activity was observed at 1000 ppm concentration against *H. armigera* and *S. litura*, respectively. Becher et al. reported that 12-epi-Hapalindole J isonitrile isolated from soil bacterium showed 100% larvicidal activity against *Chironomus riparius *[13]. Three different strains of *B. thuringiensis* showed larvicidal activity ranging between 62% and 96% against *Spodoptera frugiperda* and 100% against *Anticarsia gemmatalis *[14]. In this study some adults emerged and were small in size with varied abnormalities. 20% of the world’s top-selling pharmaceuticals and their and derived compounds were polyketide in nature and thus represent one of the most important classes of natural products, with combined revenues of over $18 billion per year [15]. Microbial polyketides are synthesized by serialized reactions of a set of enzymes called PKS with extraordinary structural diversity and an irregular distribution between strains and species, and they have been considered to play vital roles antimicrobial agents for pathogenic bacteria, fungi and also used as in pest control agents to kill insects and pests [16]. Spinosyns recovered from microorganism showed potent insecticidal activities against many commercially significant species that cause extensive damage to crops and other plants. They also exhibit activity against important external parasites of livestock, companion animals and humans [17]. Several microbial polyketides, such as avermectins and milbemycins, have been reported as potent insecticides against various insects and parasites. Furthermore, they are believed to be the biggest selling and arguably most effective acaricides and anthelmintics currently available [18]. Of the 7000 known polyketide structures, more than 0.3% has been commercialized [19]. Given the importance and potential of these compounds, the discovery of microbial polyketides has drawn increasing attention.

## Conclusions

In conclusion, polyketide metabolite showed good antifeedant, larvicidal, pupicidal and growth inhibitory activities against *H. armigera* and *S. litura*. The results indicated that polyketide metabolite would be a potential insecticide. This study is the first report on antifeedant, larvicidal, pupicidal and growth inhibitory activities against *H. armigera* and *S. litura*. This metabolite could be used for the development of new insecticidal formulation for the management of field pests.

## Methods

### Isolation and identification of *Streptomyces* sp. AP-123

**Figure 2:**
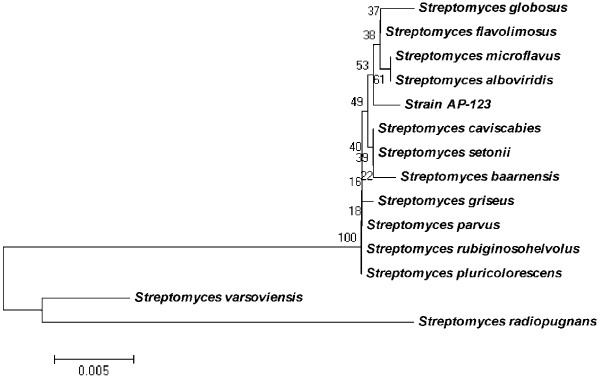
**Phylogenetic tree based on 16S rDNA gene sequence showing the relationship between *Streptomyces *sp. AP-123 and species belonging to the genus *Streptomyces *was constructed using the neighbour-joining method.** Bootstrapping values >50 are not mentioned [10].

*Streptomyces* sp. AP-123 was isolated from Andra Pradesh coast of the Bay of Bengal, India. The 16S rDNA gene (accession number JQ283107) based phylogenetic affiliation was determined by using bioinformatics tools identified *Streptomyces* sp. AP-123 as *Streptomyces* sp. with 99% sequence similarity to *Streptomyces flavogrecius* (Figure 2).

### Isolation and identification of polyketide metabolite

Isolation of polyketide metabolite and its identification have already been described in our earlier manuscript [10].

### Insect culture collection and monitoring

Larvae of *S. litura* and *H. armigera* were collected from the farmers’ field in Kancheepuram district, Tamil Nadu. Insects were cultured by following the methods of Basker et al. [20]. Briefely, the collected *H. armigera* larvae were reared individually in a plastic container (vials) and fed regularly with lady’s finger, *Abelmoschus esculentus* L. (*Malvaceae*) and *S. litura* larvae were reared on castor leaves and were kept till the larvae became pupae under the laboratory conditions (27 ± 2°C and 74 ± 5% relative humidity). The sterile soil was provided for pupation. After pupation, the pupae were collected from the soil and placed in inside the cage for emergence of adults. Cotton soaked with 10% honey solution (Dabur Honey, India) mixed with a few drops of multi-vitamins (Hi-Media, Mumbai) was provided for adult feeding to increase the fecundity. Potted cowpea plants were kept for *H. armigera* and groundnut plants were provided for *S. litura* separately inside the adult emergence cages for egg laying. After hatching, the larvae were collected from the cage and fed with standard artificial diet as recommended by Koul et al. [21] for *H. armigera*. Castor leaf was provided for *S. litura*.

### Antifeedant activity of the polyketide metabolite

Antifeedant activity of polyketide metabolite was evaluated using leaf disc no-choice method described by Basker et al. [20]. Briefly, fresh young cotton (*H. arigera*) and castor (*S. litura*) leaves were collected and cleaned thoroughly with water to remove the dust and other particles and then wiped with cotton to remove the moisture content, after that leaf discs of 4 cm diameter were punched using cork borer. Four different concentrations of the isolated metabolite such as 125, 250, 500 and 1000 ppm were evaluated in this study. The leaves disc were dipped into the metabolite for 15 min. Acetone (Thermo Fisher Scientific India Pvt. Ltd, Mumbai, India) was used as negative control since acetone was used to dissolve the compound and leaf discs dipped in azadirachtin (40.86% purity, obtained from EID-Parry India Ltd., Chennai) was used as positive control. In each plastic petridish (1.5 × 9 cm) wet filter paper was placed to avoid early drying of the leaf discs. Third instar larva of the respective insects was introduced into each petriplates. Progressive consumption of treated and control leaves by the larvae after 24 h was assessed using Leaf Area Meter (Delta-T Devices, Serial No. 15736 F 96, UK). Leaf area eaten by larvae in treatment was corrected from the negative control. Each concentrations were maintained as five replicates with 10 larvae per replicate (total, N = 50). The experiment was performed at laboratory conditions (27 ± 2°C) with 14:10 photoperiod and 75 ± 5% relative humidity. Antifeedant activity was calculated according to the formula of Bentley et al. [22]*.*

### Larvicidal activity of the polyketide metabolite

Larvicidal activity was studied using leaf disc no-choice method Basker et al. [20]. Briefly, fresh cotton and castor leaf were obtained from the garden was used in this study. After cleaning the leaves with water leave discs were made and dipped in different concentrations of the compound and assayed as mentioned in antifeedant experiment. After 24 h the larvae were continuously maintained on the untreated fresh cotton and castor leaves for *H. armigera* and *S. litura*, respectively. Insect diet was changed every 24 h. Larval mortality was observed and recorded after 96 h of treatment. Five replicates were maintained for each treatment with 10 larvae per replicate (total N = 50). The laboratory conditions were maintained as same as in the antifeedant experiment. Percent mortality was calculated according to Abbott [23].

### Pupicidal activity of the polyketide metabolite

The larvae which survived were continuously fed with normal diet as specified in larvicidal activity until they became pupae and adults. Pupicidal activity was calculated by subtracting the number of emerging adults from the total number of pupae.

### Larval and pupal durations

The survived larvae in the treatments were reared on fresh untreated leaves and their larval duration after the treatment was recorded. Pupal period was calculated from the day of pupation to the day of adult emergence.

### Statistical analysis

The data related to antifeedant, larvicidal and pupicidal activities and larval–pupal durations were analysed by one way Analysis of Variance. Significant differences between treatments were determined using Tukey’s multiple range tests (P ≤ 0.05). Probit analysis was done to calculate median lethal concentration (LC_50_) and LC_90_ using SPSS 11.5 version software package [24].

## Competing interests

The authors declare that they have no competing interest.

## Authors’ contributions

Conceived and designed the experiments: MVA NAA-D VD. Performed the experiments: MVA VS VD CM. Analyzed the data: MVA NAA-D VD CM. Wrote the manuscript: MVA NAA-D VD SJK. All authors read and approved the final manuscript.

## Acknowledgments

The authors are grateful to global Research Centre for Biotechnology, Taramani, Chennai, India, Entomology Research Institute, Loyola College and CNU for carrying out this work. Authors are thankful to Addiriyah Chair for Environmental Studies, Department of Botany and Microbiology, College of Science, King Saud University, Riyadh-11451, Saudi Arabia for financial assistance. 
